# A novel negative-pressure suction tube for emergency endoscopic hemostasis of upper gastrointestinal bleeding

**DOI:** 10.1055/a-2934-8520

**Published:** 2026-08-20

**Authors:** Jianguang Zhang, Ruozi Pan, Dizhou Fan, Bin Yang, Zhan Ma, Limei Cai, Peng Zhang

**Affiliations:** 1 Department of GastroenterologyXiong'an Xuanwu HospitalXiong'an New AreaChina; 2 Zhengzhou Dihong Electronic Technology Co., Ltd.ZhengzhouChina

Emergency endoscopic hemostasis is critical for acute upper gastrointestinal bleeding. However, retained gastric blood, clots, and food residues frequently obscure the endoscopic field and delay the identification of the bleeding source 1​ ( Fig. 1 ). Suction through a working channel of a conventional endoscope and standard gastric tubes is often inadequate for removing large clots.

**Fig. 1:**
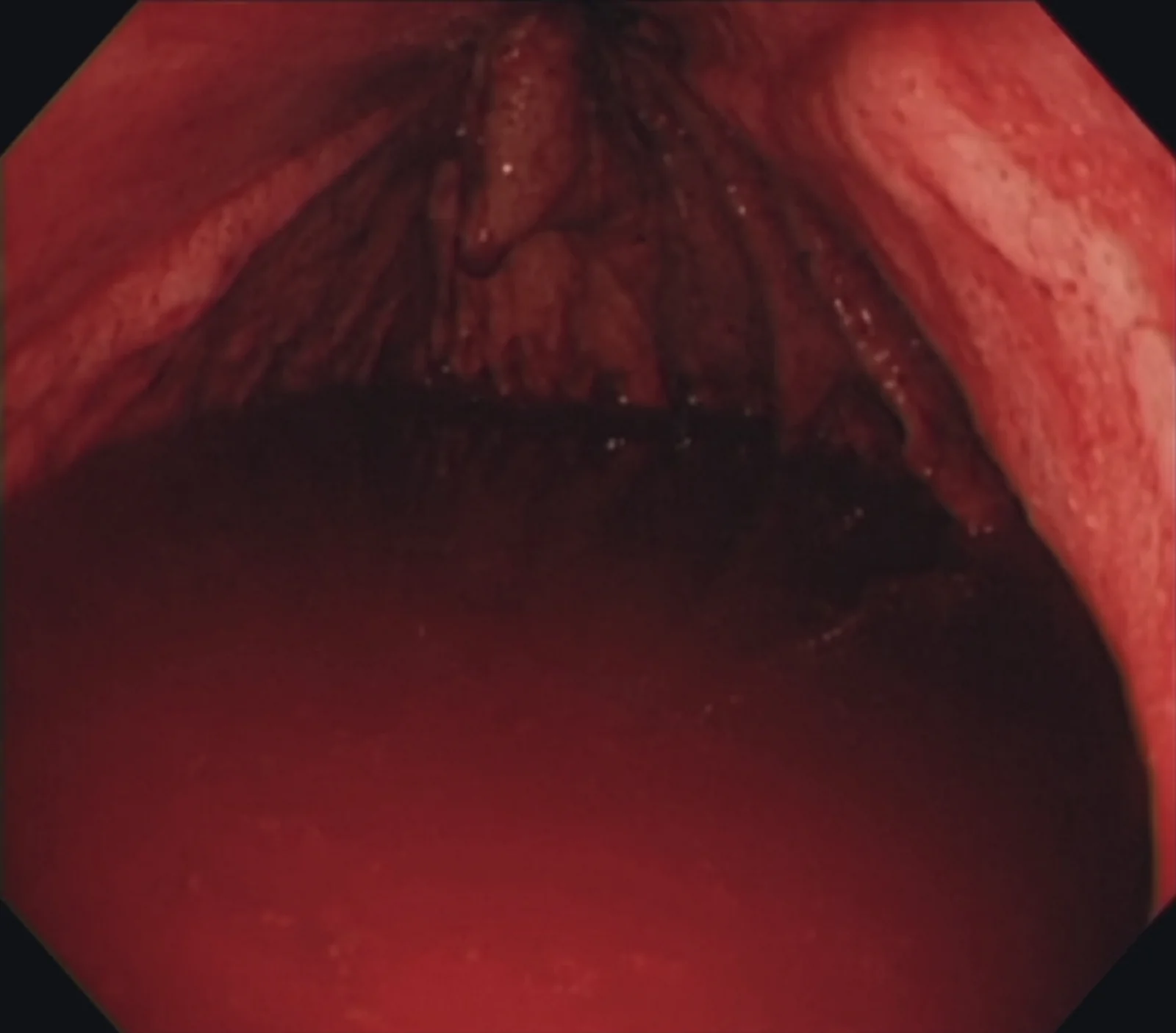
An endoscopic view showing a large amount of retained blood and clots obscuring the gastric mucosa.

We developed a novel negative-pressure suction tube (Patent No. 221731762 U) for ultrathin endoscope-guided clot evacuation ( Fig. 2 ; Video 1 ). Its main tube functions as an overtube; the lateral suction branch connects to a negative-pressure source, and sealing the elastic access port after endoscope withdrawal establishes a closed suction circuit. The distal balloon reduces mucosal suction, while finger occlusion of the control hole enables pulsed aspiration.

**Fig. 2:**
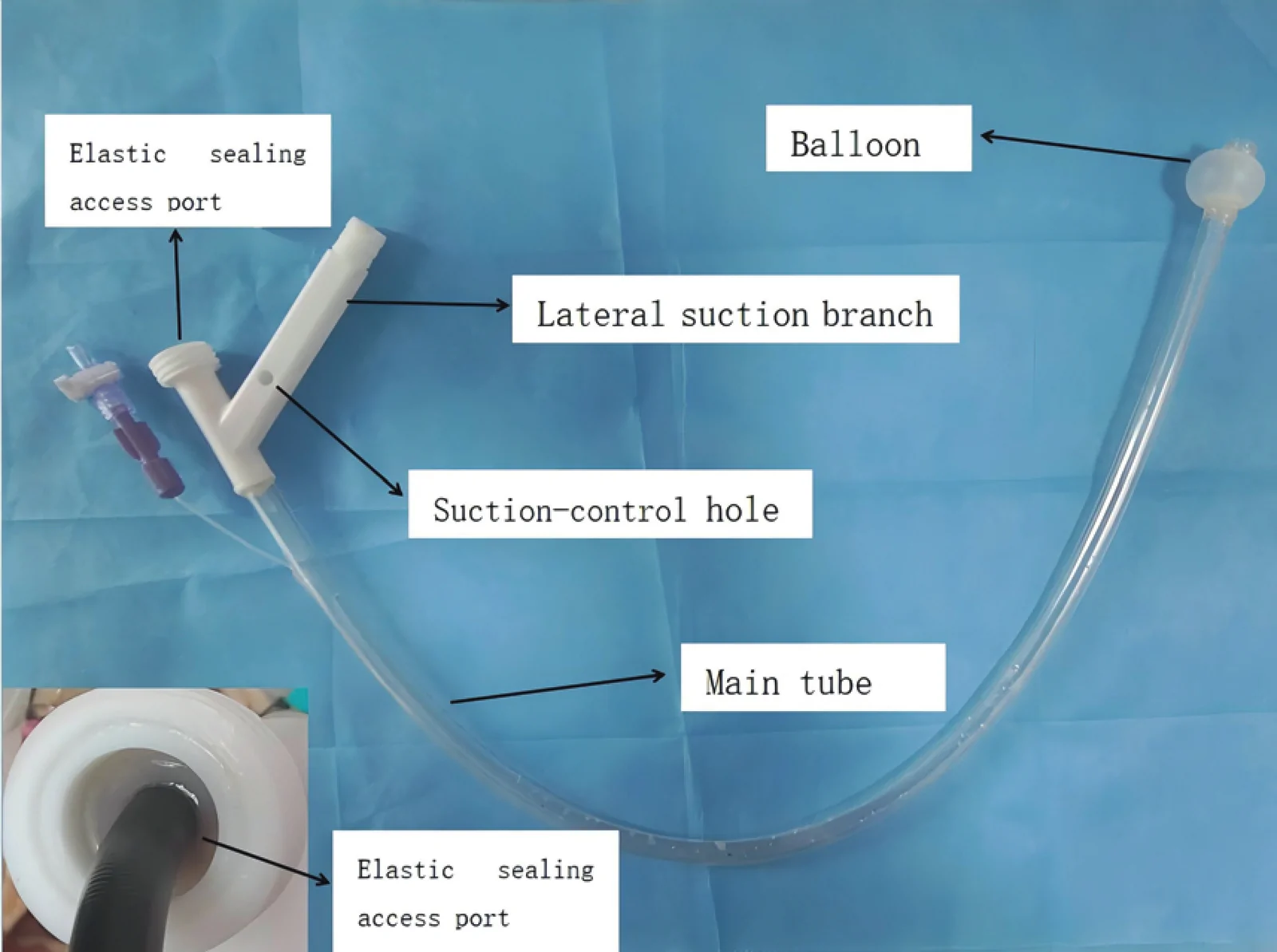
The negative-pressure suction tube. The main components are labeled as follows: a main tube, a lateral suction branch, a balloon, a suction-control hole, and an elastic sealing access port.

**Video 1** Ex vivo demonstration of the negative-pressure suction tube as an overtube, including balloon inflation, access-port sealing, pulsed suction, and evacuation of retained blood and clots. Video URI:


A closed ex vivo porcine stomach model was used to simulate active upper gastrointestinal bleeding. Each experiment involved injection of 500 mL of porcine blood containing clots. Standard suction through a 3.2-mm working channel caused repeated obstruction, leaving approximately 200 mL of clotted blood after 10 minutes. A traditional 28 F gastric tube also blocked repeatedly, leaving approximately 300 mL of clotted blood.

For the novel device, the ultrathin endoscope and overtube were advanced together ( Fig. 3 ). After positioning, the endoscope was withdrawn, the access port was sealed, and the balloon was inflated with 10 mL of air. Pulsed suction rapidly evacuated blood and clots. Residual adherent clots were identified endoscopically ( Fig. 4 ) and removed by targeted irrigation, tube repositioning, and repeat suction.

**Fig. 3:**
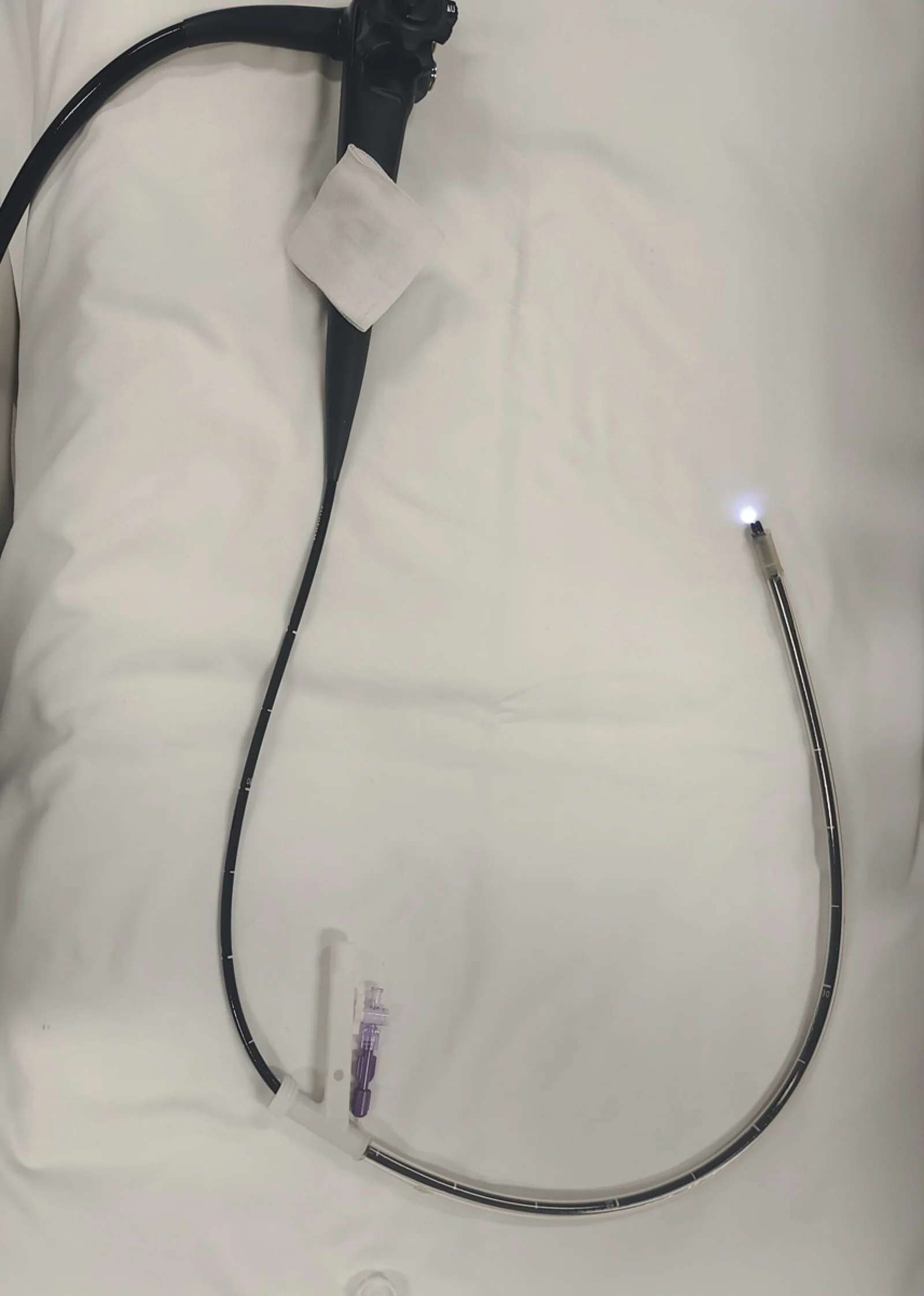
The ultrathin endoscope was inserted through the main tube, which functioned as an overtube, before both were advanced into the ex vivo porcine stomach.

**Fig. 4:**
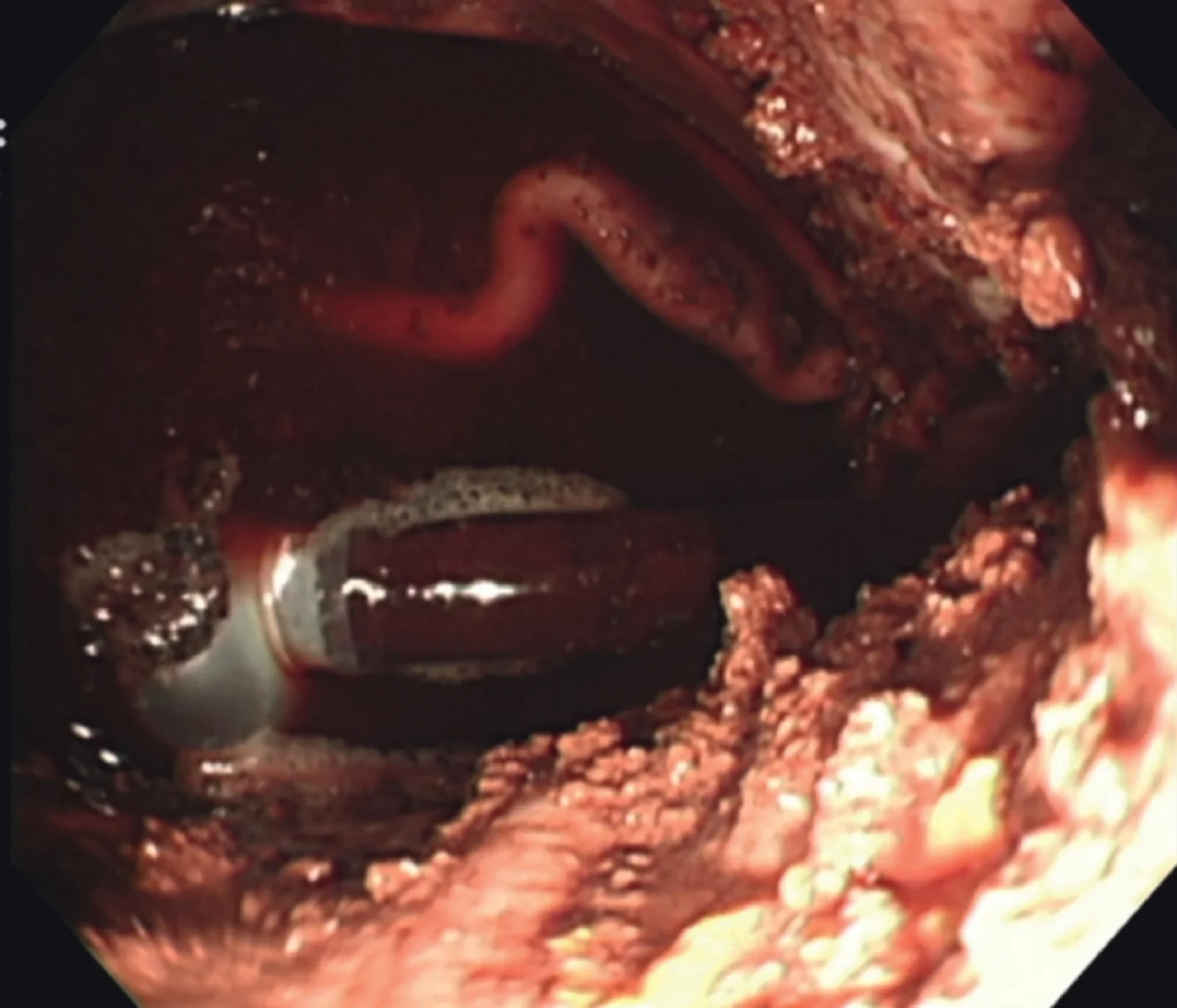
Endoscopic reassessment after initial suction showing the marked clearance of retained blood, with only a small amount of residual clot adherent to the gastric wall.

Complete clot evacuation restored clear mucosal visualization ( Fig. 5 ). A bench-tested standard-gastroscope prototype required an outer diameter exceeding 15 mm, limiting maneuverability; further miniaturization is needed before clinical evaluation. The device may be useful when conventional suction is ineffective during emergency hemostasis. 2​ Further clinical studies are required.

**Fig. 5:**
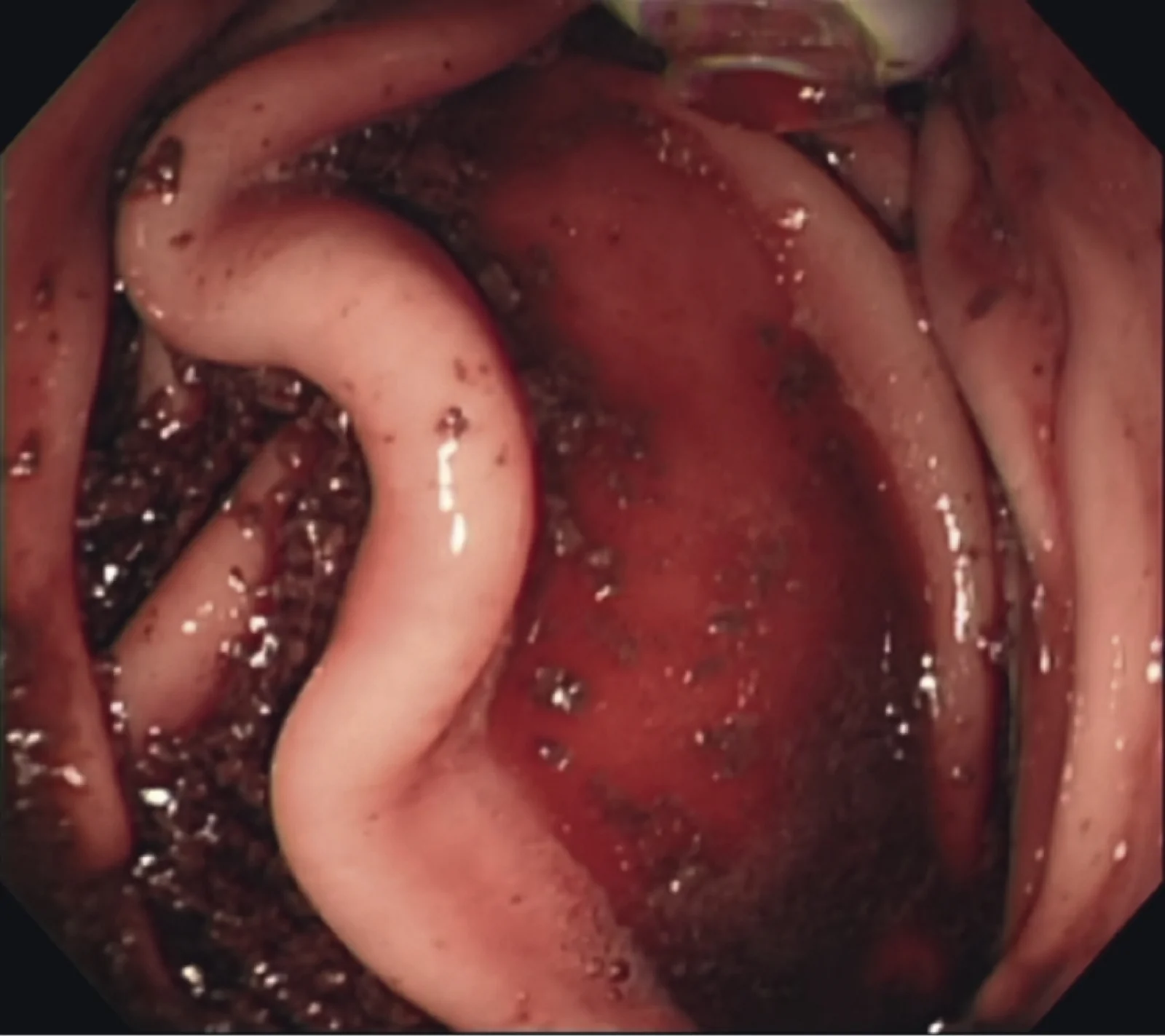
A final endoscopic view after irrigation and repeat suction, showing the complete clearance of clots and clear visualization of the gastric mucosa.

Endoscopy_UCTN_Code_TTT_1AO_2AN

## References

[1] Gralnek I M Stanley A J Morris A J Endoscopic diagnosis and management of nonvariceal upper gastrointestinal hemorrhage: European Society of Gastrointestinal Endoscopy Guideline — Update 2021 Endoscopy 2021 53 300 332 33567467 10.1055/a-1369-5274

[2] Laine L Barkun A N Saltzman J R Martel M Leontiadis G I ACG Clinical Guideline: Upper Gastrointestinal and Ulcer Bleeding Am J Gastroenterol 2021 116 899 917 33929377 10.14309/ajg.0000000000001245

